# Relationship between IL-17 serum level and ambulatory blood pressure in women with polycystic ovary syndrome

**DOI:** 10.15171/jnp.2017.04

**Published:** 2016-08-08

**Authors:** Fatemeh Foroozanfard, Alireza Soleimani, Elham Arbab, Mansooreh Samimi, Mohammad Reza Tamadon

**Affiliations:** ^1^Department of Gynecology and Obstetrics, Kashan University of Medical Sciences, Kashan, Iran; ^2^Department of Internal Medicine, Kashan University of Medical Sciences, Kashan, Iran; ^3^Department of Internal Medicine, Semnan University of Medical Sciences, Semnan, Iran

**Keywords:** IL-17, Polycystic ovary syndrome, Inflammatory factors, Renin-angiotensin system

## Abstract

**Background:**

Polycystic ovary syndrome (PCOS) is one of the most common endocrine disorders with an inflammatory basis. It is associated with hyperandrogenism in women and can be also associated with increased activity of the renin-angiotensin system (RAS). Approximately 5% to 10% of women of reproductive age are affected by this disease. This syndrome is the main cause of infertility. Blood pressure may be one of the complications of the syndrome.

**Objectives:**

In this study, we sought to assess the role of the IL-17 inflammatory cytokine in increasing blood pressure in patients with PCOS.

**Patients and Methods:**

In this cross-sectional study, after obtaining informed consent, we evaluated 85 patients with PCOS. IL-17 serum level was measured after separating the serum via ELISA method. The results obtained for the two groups of patients with high blood pressure and normal blood pressure were compared with each other.

**Results:**

The daytime blood pressure was abnormal in eight patients, while it was normal in 72 patients. The blood pressure during the day had a direct correlation with the IL-17serum level; as a result, the mean IL-17 serum level in patients with high blood pressure was 77.10 ± 17.94 ρ g/ml while in those with normal blood pressure it was 55.20 ± 13.71 ρ g/ml (*P* = 0.001). High blood pressure during the night also showed a direct relation with theIL-17 serum level (*P* = 0.001). In addition, increasing of ambulatory 24-hourblood pressure was significantly related with IL-17 serum level, in such a way that the IL-17 serum level of people with high blood pressure rose by almost 22 ρg/ml during 24 hours (*P* = 0.001).

**Conclusions:**

Our results showed an association between PCO syndrome and inflammatory factors. The IL-17 serum level was directly associated with the increase in blood pressure.

**Implication for health policy/practice/research/medical education:**

This study has implication for the researcher as well as practitioners, gynecologist and internist.

## 1. Background

Polycystic ovary syndrome (PCOS) is one of the most common endocrine disorders which is associated with increased activity of the renin-angiotensin system (RAS) and hyperandrogenism in women. Approximately 5% to 10% of women of reproductive age (12-45 years old) are affected by this disease. This syndrome is considered to be one of the main causes of infertility (1-4). The results of previous studies indicate that at least 75% of patients with PCOS have the symptoms of insulin resistance syndrome (IRS) (5). All the patients with PCOS do not have all the abnormalities and clinical syndromes of IRS; however the risk of insulin resistance in PCOS patients is high. As a result, it is necessary to study the relationship between these two complications. The need for studying this issue is more highlighted when we bear in mind that every IRS symptom is a risk factor for cerebrovascular accident (6). According to the American College of Endocrinology, the high blood pressure over 130/85 mm Hg is one of the indicators of IRS (7). Overall, the incidence of hypertension in patients with PCOS is higher than those of the same age without the disease (8-10). According to the results of a study, ambulatory (24-hour) blood pressure monitoring showed high systolic blood pressure during the day, even after the change in body mass index (BMI). It also showed higher levels of insulin sensitivity and body fat distribution, compared to healthy subjects (11). Due to the presence or potential risk of cardiovascular accident, patients with PCOS should be monitored for blood pressure control (7). In fact, blood pressure is the most important modifiable risk factor which can lead to morbidity and mortality in cardiovascular, cerebrovascular, and renal diseases worldwide (12).

There are strong evidence on the role of androgens in the development of hypertension in patients with PCOS (13). The mechanism of this effect is unknown, however it has been shown that the control of RAS in female animal subjects, whose ovaries are removed, can prevent androgen supplements to increase blood pressure. Such data indicate that RAS components play a role in the increase of blood pressure caused by androgens (14). Probably, androgens can increase blood pressure in patients with PCOS via increasing the expression of RAS components (15).

The clinical condition of hypertensive-associated vascular disease is an inflammatory process, in which chronic inflammation plays a key role in the pathophysiology of hypertension (16). This inflammatory process involves a series of interactions between inflammatory cells and cytokines; in addition, the reactive oxygen species (ROS) and RAS together with increased local production of angiotensin II (AngII) play a major role in this process (17). It has been reported that AngII, as a major mediator of oxidative stress, increases vascular smooth muscle tone. The presence of AngII in vascular smooth muscle cells and endothelium of hypertensive patients, not only increases the expression of chemokine MCP-1, IL-6, and IL-8, but also activates NFκB which is an important transcription factor in the inflammatory process; it also activates endothelin-1 (ET-1) which is an important mediator in chronic inflammatory vascular processes. AngII also causes diapedesis (leukocyte extravasation) in inflammatory cells. It also increases the TGF-β signaling which is one of the main factors involved in the pathogenesis of cardiovascular disorders, including hypertension (18). Based on the findings of clinical and laboratory studies, other components of the RAS, including aldosterone and/or mineralocorticoid receptor (MR) can also lead to oxidative stress and vascular inflammation (19); as a consequence, the administration of aldosterone and MR stimulants increase the serum level of IL-6 and IL-12 (20).

Nowadays, there are many evidences suggesting the role of IL-17 in inflammation of tissues. IL-17 acts via the induced release of pro-inflammatory cytokines and other cytokines which stimulate neutrophils (21). IL-17 cytokine, as the main cytokine secreted by the Th17 cells, increases the activity of T cells and induces endothelial secretion of several pro-inflammatory mediators, including IL-1, IL-6, tumor necrosis factor-α (TNF-α), NOS-2, metalloproteinase, and chemokines; as a consequence, it enhances endothelial inflammation (21,22). In addition, IL-17 causes hypertension and vascular dysfunction induced by AngII and it can be considered as a target for treatment of this disease (23).

Since at least three different main pathways (Th1 / IL-12 / INF-γ, Th2 / IL-4-IL-5 / IL-13 and Th17/IL-17) are involved in the inflammatory responses, the identification of the major immunological pathway responsible for the development of any kind of inflammatory disease can play a potential important role in the treatment of the disease. Hence, any medical and therapeutic measure aimed to inhibit IL-17 cytokine can greatly help to treat various inflammatory responses (24). So far, numerous studies have been conducted to determine the effect of different inflammatory cytokines on patients with PCOS. For example, in the study by Kaya et al in 2010, they tried to determine the relationship between the levels of IL-6 and C-reactive protein (CRP) inflammatory markers and fibrinogen and nitric oxide and ambulatory blood pressure in patients with PCOS. The results showed that the increased levels of IL-6 were likely associated with 24-hour systolic and diastolic blood pressure in the studied patients (25).

According to the mentioned above findings, it is logical to assume that the activation of Th17/IL-17 pathway is at least one of the factors causing the inflammatory process in Hypertensive-associated vascular disease in patients with PCOS. It probably acts through stimulating the RAS. As a result, given the need to control hypertension in patients with PCOS (6), the control of IL-17 can be a valuable treatment target in this case.

## 2. Objectives

To confirm the above mentioned hypothesis, in this study we tried to investigate the possible association between increased IL-17 serum level and 24-hour blood pressure in patients. Compared with the conventional measurement methods, the 24-hourambulatory measurement provides more reliable and accurate data about blood pressure, especially in individuals with hypertension (25). This study aimed to further investigate the immunopathogenic aspects of hypertension in patients with PCOS to facilitate the identification of treatment goals.

## 3. Patients and Methods

### 3.1. Study patients

In this cross-sectional study, after obtaining an informed consent, 85 patients with PCOS were enrolled to the study. Based on the inclusion criteria, patients with PCOS must meet at least two out of the three following criteria;

A) Polycystic ovary confirmed by ultrasound
B) Oligomenorrhea (cycle over 35 days, or amenorrhea, the absence of menstruation for three consecutive months)
C) Clinical hyperandrogenism (hirsutism with modified Ferriman-Gallwey score=5, or hyperandrogenemia when level of prolactin, TSH, 17-hydroxyprogesterone were normal and there is no androgen secreting tumor).

Exclusion criteria were the followings; hypothyroidism, hyperprolactinemia, androgen secreting tumor, non-classic adrenal 21-hydroxylase deficiency, chronic kidney injury, congestive heart disease, confirmed diabetes mellitus, chronic hypertension, acute illness, all autoimmune and non-autoimmune inflammatory diseases, different types of cancer, and patients^’^ unwillingness to continue participation in the study.

### 3.2. Assessments

For all the studied subjects; BMI and levels of fasting blood sugar* (*FBS*),* thyroid stimulating hormone (TSH), prolactin*,* follicle-stimulating hormone (FSH), luteinizing hormone (LH), dehydroepiandrosterone sulfate (DHEAS), triglyceride (TG), cholesterol, low-density lipoprotein-cholesterol (LDL-C), high-density lipoprotein-cholesterol (HDL-C), CRP, insulin, free testosterone, and 17-hydroxyprogesterone were measured. The 24-hour ambulatory blood pressure monitoring was performed by every patient at regular time intervals via using a mobile device. Blood pressure was measured during the day and at night separately. A blood pressure over 125/75 mm Hg during the night and over 140/90 mm Hg during the day and an overall blood pressure over 135/85 mm Hg were considered abnormal. When a patients’ blood pressure was 15% above the normal values, the condition was considered as abnormal. In addition, when a patient’s blood pressure over the night did not lower by 15% relative to the day time blood pressure, the condition was also considered as abnormal.

IL-17 serum level was measured after separating the serum via ELISA method and using USCN kit made in the United Kingdom with a catalog number of E90384Hu and Lot number of L120605095.

After collecting the data, the results obtained for the two groups of patients with high and normal blood pressure were compared with each other. First, the statistical indicators of IL-17 serum level, blood pressure, and demographic factors (BMI, age) were calculated.

### 3.3. Ethical issues

1) The research followed the tenets of the Declaration of Helsinki; 2) informed consent was obtained, and they were free to leave the study at any time and 3) the research was approved by the ethical committee of Kashan University of Medical Sciences.

### 3.4. Statistical analysis

Pearson’s correlation coefficient or Spearman’s test were used to assess the correlation between systolic and diastolic blood pressure and the mentioned factors. In addition, multiple regression analysis was conducted to examine the association between IL-17serum level and blood pressure; it was also used to adjust for potential confounding variables such as age and BMI.

## 4. Results

A total of 85 patients were enrolled in the study. The mean age of the patients was 26.31 ± 4.1 years, ranging from of 17 to 39 years. The mean IL-17 serum level was 57.3 ± 15.6 ρg/ml. The data of patients illustrated in tables 1 to 3.

The mean BMI in the studied subjects was 27.65 ± 5.05 kg/m^2^ which was higher than the normal range. However, this index did not show a significant relationship with IL-17 serum level (*P*=0.05). On the other hand, the FBS levels in the studied patients was 88.48 ± 19.14 mg/dl, which did not show a significant relationship with IL-17 serum level (*P*=0.25). Around 16 patients had high insulin levels; however, this hormone did not show a significant relationship with IL-17 serum level (*P*=0.21) (Table 1).

**Table 1:** Mean ± SD of variables and their association with serum levels IL-17 in patients with PCOS

**Variables**	**Mean ± SD**	**P value**
IL-17 (ρg/ml)	57.33 ± 15.60	-
BMI (kg/m^2^)	27.65 ± 5.5	0.05
Age (years)	26.31 ± 4.10	0.57
FBS (mg/dl)	88.48 ± 19.14	0.25
TSH (micIU/ml)	2.41 ± 1.41	0.27
PRL (ng/ml)	56.8 ± 124	0.75
FSH (mIU/ml)	4.59 ± 6.26	0.25
LH (mIU/ml)	7.5 ± 3.39	0.2
DHEASO4 (µg/ml)	2.64 ± 3.13	0.24
TG (mg/dl)	43.03 ± 1.41	0.7
Cholesterol (mg/dl)	27. 23 ± 1.60	0.83
Fasting insulin (µIU/ml)	8.6 ± 7. 29	0.21
FT (ng/ml)	2.49 ± 7. 27	0.27
HP17 (ng/dl)	1.3 ± 0.91	0.49
LDL (mg/dl)	99.23 ± 26. 25	0.97
HDL (mg/dl)	46.88 ± 1. 9	0.73
LDL/HDL ratio	2.2 ± 0.66	0.63

The daytime blood pressure was high in eight patients while it was normal in 72 patients. The daytime blood pressure value was associated with IL-17 serum levels. There was an increase in daytime blood pressure which was correlated with increasing the IL-17 serum levels. The results showed that, the mean IL-17 serum level in patients with high blood pressure was 77.10 ± 17.94 ρg/ml while in those with normal blood pressure it was 55.20 ± 13.71 ρg/ml, which showed a statistically significant increase (*P*=0.001) (Table 2). Moreover, nighttime blood pressure showed a significant relationship with IL-17 serum levels. Accordingly, with an increase in blood pressure, the IL-17 serum level was also increased (*P*=0.001). In addition, the increase in ambulatory 24-hour blood pressure was significantly related with IL-17 serum levels. Our study showed that, IL-17 serum level of people with high blood pressure rose by almost 22 ρg/ml during 24 hours (*P*=0.001).

**Table 2:** Comparison of ambulatory blood pressure monitoring changes in various conditions with serum IL-17 levels in patients with PCOS

**Blood pressure (mm Hg)**	**Mean ± SD**	**P value**
Daytime blood pressure	77.10 ± 17.94	0.001
	55.20 ± 13.71	
Nighttime blood pressure	74.23 ± 15.53	0.001
	53.82 ± 13.07	
Ambulatory 24-hour blood pressure	76.78 ± 14.31	0.001
	54.96 ± 13.94	
More than 15% reduction of blood pressure over the night compared with the day	57.04 ± 16.84	0.94
	57.37 ± 15.54	

According to the results, BMI was 31.56 ± 4.69 kg/m^2^ in people with high ambulatory blood pressure while it was 27.10 ± 4.69 kg/m^2^ in those with normal ambulatory blood pressure. With an increase in blood pressure level, BMI was increased too (*P*=0.009) (Table 3).

**Table 3:** Association of association of various variables with ambulatory blood pressure monitoring in patients with PCOS

**Variables**	**Ambulatory blood pressure**	
	**Mean ± SD**	**P value**
BMI (kg/m^2^)	27.1 ± 4.69	0.009
	31.56 ± 4.69	
Age (Years)	26.35 ± 4.27	0.55
	27.22 ± 2.63	
FBS (mg/dL)	88.19 ± 8.78	0.8
	89 ± 12.51	
TSH (micIU/ml)	2.52 ± 1.34	0.09
	1.73 ± 1.25	
PRL (ng/ml)	63.6 ± 134.47	0.33
	19.51 ± 12.54	
FSH (mIU/ml)	4.86 ± 6.81	0.42
	3.04 ± 0.67	
LH (mIU/ml)	7.71 ± 3.58	0.12
	5.79 ± 1.92	
DHEASO4 (µg/ml)	2.6 ± 3.6	0.55
	3.27 ± 4.35	
TG (mg/dl)	46.1 ± 1.4	0.73
	22.59 ± 1.46	
Cholesterol (mg/dl)	27.54 ± 1.6	0.93
	28.77 ± 1.56	
Ins (µIU/ml)	8.23 ± 7.59	0.9
	7.9 ± 6.39	
FT (ng/ml)	2.59 ± 3.35	0.76
	2.23 ± 3.69	
HP17 (ng/dl)	1.01 ± 0.8	0.28
	1.36 ± 1.69	
HDL (mg/dl)	47.15 ± 1.13	0.6
	45 ± 12.9	

## 5. Discussion

The mean IL-17 serum level in the studied patients was 57.33 ± 15.6 ρg/ml. The BMI of the patients was also high which was in an agreement with the results of a study by Elting et al in 2001 (26). On the other hand, the BMI of the patients was associated with IL-17 serum levels. While, with increasing the BMI, the IL-17 serum level increased too. However, the observed relationship was not statistically significant. This finding, i.e. the simultaneous increase in BMI and an inflammatory cytokine, is in accordance with the findings of Yang et al (27) in 2011. They showed that, IL-18 serum levels was higher in overweight patients. In their study, IL-18 serum level had a direct positive relationship with BMI, testosterone, and insulin resistance (27). Taking into consideration the inflammatory cytokines, the results of a study Wu et al in 2007 regarding TNF-α inflammatory cytokines is in agreement with our results, while their findings about IL-6 inflammatory cytokine is inconsistent with our results. In their study, high BMI had a significant relationship with increased TNF-α and decreased level of IL-6 in the follicle cells (28). Likewise, in the study conducted by Knebel et al, IL-8 and IL-17 did not change in patients, but other cytokines such as IL-1B, IL-6, and TNF-α increased. It was also found that the level of IL-17 cytokine was decreased in cases, compared with obese people in the control group (29). Although several studies have shown an increase in inflammatory markers in patients with PCOS, but it is not clear whether the increase of immune cells and molecules is associated with diseases or is due to obesity and high BMI.

Another important finding in our study was no significant association of FBS and insulin levels in patients with PCOS with levels of IL-17. Although insulin level had increased to some extent, but this increase was not associated with IL-17. Considering other inflammatory cytokines, our results was contradictory with the results of some other studies. For example, some studies have shown that IL-18 cytokine is closely associated with insulin resistance and metabolic syndrome, including high blood pressure, hyperlipidemia, diabetes mellitus, obesity, and hypertension (30,31). Moreover, other studies have also shown that an increase in leukocytes in women with PCOS is closely associated with insulin resistance through homeostasis model (32-34).

In our study, serum TSH level was normal in all the studied patients and we did not observe any increase or decrease in thyroid function. In addition, prolactine and FSH levels were normal in all the patients and they did not have any significant relationship with IL-17 serum level. In our study, LH levels increased only in three patients and its relationship with serum IL-17 was not significant. Considering other inflammatory cytokines, our results are inconsistent the results of a study by González et al in 2010. They showed that the macrophage migration inhibitory factor (MIF) chemokine macrophage in patients was significantly higher than that in the control group and its level was directly associated with the trunk obesity, CRP, LH, testosterone, and androstenedione (35). In addition, other studies have shown that, compared with normal healthy people, women with PCOS have lower levels of the FSH and SHBG (sex hormone binding globulin) and high density lipoprotein (36-39).

In this study, the levels TG and cholesterol were high in five patients. There was no significant relationship between IL-17 serum level and TG-C, cholesterol, LDL-C, and HDL-C levels. This finding is inconsistent with the results of a study by González et al. They showed that the IL-6, sICAM-1, CRP, PAI-1, systolic and diastolic blood pressure, TGs, fasting insulin, and insulin resistance (HOMA-IR) levels were higher in patients with PCOS than in the controls. MCP-1 chemokine was associated with NFκB activation, hyperglycemia, and increased androstenedione. In addition, the levels of sICAM-1, IL-6, and CRP were higher in patients with PCOS who were obese (40). In addition, other studies have shown that, compared with healthy individuals, patients with PCOS have higher levels of testosterone, insulin, cholesterol, TG, and luteinizing (36-39).

In this study, we found that the ambulatory 24-hour blood pressure during the day and night had a significant relationship with IL-17, hence, the IL-17 level was higher in patients with high blood pressure. This finding is in line with the results of a study by Kaya et al in 2010. In this study, we aimed to determine the relationship between IL-6, CRP, fibrinogen, and nitric oxide levels and ambulatory blood pressure in women with PCOS. The results showed that the increased levels of IL-6 were probably associated with 24-hour systolic and diastolic blood pressure of the patients. The study showed that the level of IL-6 in patients with high blood pressure was higher while the level of NO was lower (25). It is possible that, IL-17 cytokine as the major cytokine secreted from Th17 cells increased the activity of T cells and induced endothelial secretion of several pro-inflammatory mediators, including IL-1, IL-6, TNF-α, NOS-2, metalloproteinase, and chemokine, and thus enhanced endothelial inflammation (21,22). On the other hand it has been detected that levels of IL-6 in patients with hypertension with no history of systemic disease is higher and has a direct relationship with proportion of blood pressure (25,41-45). In addition, recent studies have also shown that increased CRP levels are associated with increased systolic and diastolic blood pressure (45-48). Given that IL-6 increases the CRP levels, IL-6 is also likely to act similarly and cause hypertension in patients with PCOS (43,49,50). Moreover, IL-17 causes hypertension and vascular dysfunction induced by AngII (23). Perhaps these two pathways are the main cause of a positive direct relationship between the IL-17 serum level and increased levels of blood pressure. According to the results of our study, there was no significant relationship between IL-17 serum level and lack of reduction in blood pressure over the night, compared with the day. This finding perhaps reflects the fact that the decreased blood pressure observed among other patients is likely due to other environmental factors, such as getting relaxed at night and staying away from environmental stress. In other words, the lack of reduction in blood pressure is not necessarily rooted in the basic inflammatory conditions of the patients.

Our study showed that the ambulatory blood pressure had a direct relationship with BMI, while an increase in BMI, blood pressure increased too. The results of this study are consistent with the results of a study by Elting et al which was conducted on women with PCOS. The mentioned study showed that blood pressure of the patients was significantly higher than the control group. On the other hand, the studied patients had significantly higher levels of BMI and weight gain (26).

In our study, insulin level in patients with high blood pressure was lower than that in patients with lower blood pressure. However, the difference was not significant. This result is inconsistent with the results of a study by Bentley-Lewis et al (51) that examined the increase in insulin resistance (as a factor rising blood pressure) compared with the increase of androgens in women with PCOS who were treated with contraceptives and metformin (used to increase insulin sensitivity). The mentioned study showed that the mean systolic and diastolic blood pressure were high in this group of patients however they had a sharp decline after taking metformin (51). The relationship between systemic hypertension and PCOS is not fully explained (52) but insulin resistance and hyperinsulinemia are among the predictors of hypertension in women with PCOS (53). So far, no study has shown a direct relationship between blood pressure and insulin resistance parameters. Despite the fact that insulin resistance is a prognostic factor for hypertension in these patients, the relationship between different levels of blood pressure in patients with PCOS and various heart risk factors has not become clear yet. In addition, there are few studies which have investigated the blood pressure in PCOS patients based on mean arterial pressure (MAP) (54,55). It is possible that, various mechanisms such as inflammatory reactions, hyperandrogenism, angiotensin-aldosterone system, and sympathetic system are interacted (51-58).

## 6. Conclusions

Considering the significant association between blood pressure level and IL-17 pro-inflammatory cytokine secreted from Th17 cells, it can be concluded that metabolic syndrome, which is a major cause of mortality and morbidity in patients with PCO, is under the influence of inflammatory factors. Therefore, the increased level of such factors can enhance the related complications in the patients. Therefore, control of inflammation and prevention of autoimmune processes by treatmental modalities and also the interruption of inflammatory cascade can affect both the disease and its complications.

## Limitations of the study

Small proportion of the patients was a limitation of our study. It is recommended to conduct further studies to determine the factors which interfere with or are associated with the immune system and other inflammatory pathways such as Th22 pathway. It is also recommended to study the protective factors and methods used to prevent the onset or exacerbation of inflammatory processes.

## Acknowledgments

This study is adapted from the M.D. thesis of Elham Arbab. The authors gratefully acknowledge from Dr. Alireza Moraveji, Dr. Hassan Nicoeinejad and Mr. Mehrdad Zahmatkesh who have otherwise contributed to the preparation of the manuscript.

## Authors’ contribution

All authors contributed equally and signed the manuscript.

## Conflicts of interest

The authors declared no competing interests.

## Funding/Support

This study was a funded thesis at Kashan University of Medical Sciences (Grant# 91087).
